# Long-term monitoring dataset of fish assemblages in rocky tidepools on the southern coast of Taiwan

**DOI:** 10.1038/s41597-022-01778-5

**Published:** 2022-10-21

**Authors:** Lin-Tai Ho, Shen-Chih Wang, Kwang-Tsao Shao, I-Shiung Chen, Hungyen Chen

**Affiliations:** 1 Institute of Marine Biology, National Taiwan Ocean University, Keelung, 20224 Taiwan; 2 National Museum of Marine Science and Technology, Keelung, 20248 Taiwan; 3 Biodiversity Research Center, Academia Sinica, Taipei, 11529 Taiwan; 4 Center of Excellence for the Oceans, National Taiwan Ocean University, Keelung, 20224 Taiwan; 5 Department of Agronomy, National Taiwan University, Taipei, 10617 Taiwan

**Keywords:** Biodiversity, Community ecology

## Abstract

Long-term data of fish assemblages collected in the rocky intertidal zone provides a valuable resource for elucidating the temporal variations in species diversity and intertidal ecosystems. In this study, we describe a long-term time-series dataset of fish collected by counting the number of anesthetized fish at sampling stations in the rocky tidepools on the southern coast of Taiwan. The species assemblages were monitored seasonally at the two stations for 16 y (2000–2008 and 2012–2018). In total, 86 samples containing 5137 individuals belonging to 82 species were recorded. Our data can be used for elucidating the temporal variations in fish assemblages and intertidal ecosystems and as background information for the resilience of the fish community conservation in coastal areas. The current study presents valuable data for researchers to understand the temporal and spatial variations in species abundance, richness, diversity, and composition in relation to climate change, environmental factors, and human activities.

## Abstract (summary)

**Table:** 

Measurement(s)	Taxonomic Identification Below Species Level
Technology Type(s)	visual observation method
Factor Type(s)	species
Sample Characteristic - Organism	Gnathostomata
Sample Characteristic - Environment	intertidal zone
Sample Characteristic - Location	Taiwan Island

## Background & Summary

Tidepools are sites for investigating the variations in fish assemblages in the unique ecosystems of rocky intertidal zones^1^. The species diversity and composition are varied depending on conditions of the tidepool^2,3^. For examples, the tidepool volume directly affects to the number of species and individuals^3^. In addition, fish assemblage is varied by the location of tidepool within intertidal zone, through the differences in degree of dissolved oxygen, wave exposure, and other local factors^4,5^. Fish assemblages in the intertidal zone on the reef coasts of the Pacific Ocean^2,5,6^, the USA^7,8^, Japanese islands^9,10^, and Western Europe^11–13^ have been studied. Many of these reports have investigated seasonal and annual changes in community structures^5,8,14,15^ and food web network^16^, and in the context of the resilience and stability of assemblages^5,17^. However, relatively few studies have been long-term surveys^7,8,18^, and there are few reports from tropical and subtropical areas^19^. In 2020, Ho *et al*.^19^ described a long-term time-series dataset of fish collected in the intertidal zone containing 10966 individuals belonging to 106 taxa over 20 y (1999–2018). This is the only published long-term temporal dataset of fish assemblages in the Taiwan intertidal zone.

Long-term temporal datasets of species abundance and richness are necessary to explore the underlying mechanisms of changes in fish assemblages and evaluate the effectiveness of conservation measures^20^. Unfortunately, investment in long-term ecological monitoring of all fish communities in Taiwan is inadequate, resulting in collected data that are mostly short-term, fragmented, incomplete, and improperly archived^21–23^. In Taiwan, the best example of a long-term marine ecological dataset is a survey of fish assemblages in the seas around the nuclear power plants of northern coast, which contains over 15 y of monthly or seasonally gathered data systematically collected by impinged fish sampling at cooling water intake screens^20,24,25^, observed by an underwater diving visual census, and collected by trammel net fish sampling near thermal discharges^26^. Temporal monitoring of projects in Taiwan is concerned with the ecological restoration of sewage pollution, cold damage, or cold-water intrusion, targeting species diversity and composition of fish communities^22,27^.

In the present study, we describe a long-term time-series dataset of fish collected by counting the number of anesthetized fish at sampling stations in rocky tidepools in the intertidal zone on the southern coast of Taiwan. Long-term data provide a valuable resource for elucidating temporal variation in fish assemblages and intertidal ecosystems. Studies on subtidal fish assemblages in Taiwan have shown that there are significant differences in species composition between the northern and southern coasts^28,29^, but there are few reports on intertidal fish. Our data can be used to understand the status and changes of species diversity in the intertidal zone of southern Taiwan, as well as differences in intertidal species composition from northern Taiwan^19^. Additionally, these data can be used as background information for the variation and resilience of fish community conservation in coastal areas. Finally, researchers can also use the data to understand temporal variations in species abundance, richness, diversity, and composition concerning climate change, environmental factors, and anthropogenic pressures.

## Methods

**Fig. 1:**
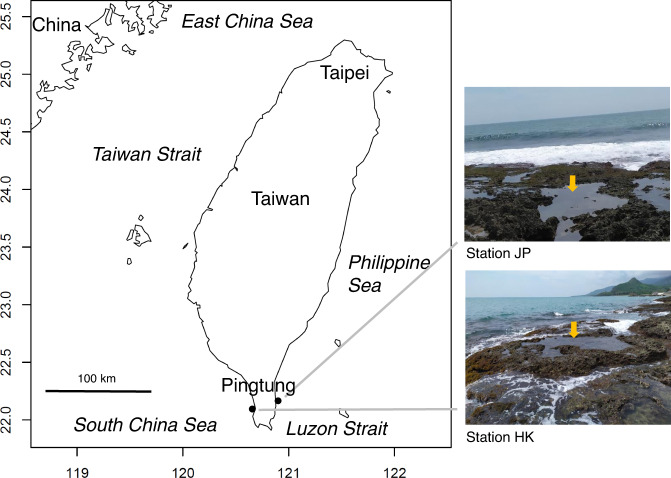
Map and photographs of sampling stations (Stations HK and JP).

**Fig. 2:**
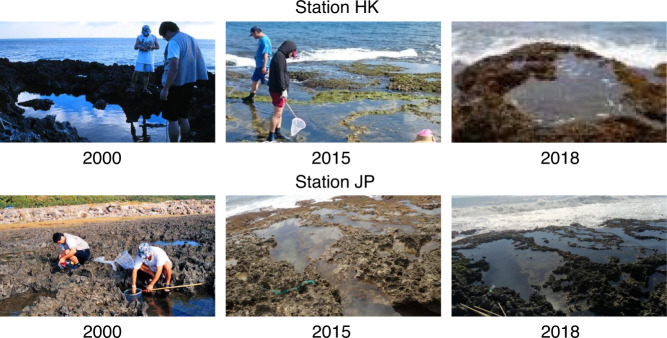
Photographs of sampling stations.

Fish community composition data were collected at two sampling stations in rocky tidepools in the intertidal zone on the southern coast of Taiwan (Figs. 1, 2). Station HK (WGS84: 22° 05′ 51″ N, 120° 43′ 25″ E) was located near Haikou in Pingtung County, and station JP (22° 08′ 18″ N, 120° 54′ 12″ E) was near Jiupeng in Pingtung County. Station HK is approximately 18 km west of station JP. Both stations were located near Kenting National Park, which provides habitat and protection from human activity for coastal fish. Besides, the stations were less disturbed by tourists due to the rough ways to the tidepools and the sharp reefs along the shore. Therefore, our samples might be suitable as time series data on long-term species diversity. The same tidepool at each station was monitored continuously during the study period, and only one sample was collected at each station per sampling. The tidepools were located within the coral reef area, and the coastal and underwater topographies, substratum type, tidal range, and wave exposure at both tidepools were very similar to each other. The tidepools were completely flushed at high tide. The locations of the tidepools were selected to be very close to the low tide line, but not completely submerged at high tide. The tidepools were selected based on the need to form an enclosed area and maintain a proper water depth at low tide, with seawater exchange between high and low tides. In addition, the tidepools with sufficient caves and cracks for the fish to hide in were selected.

**Fig. 3:**
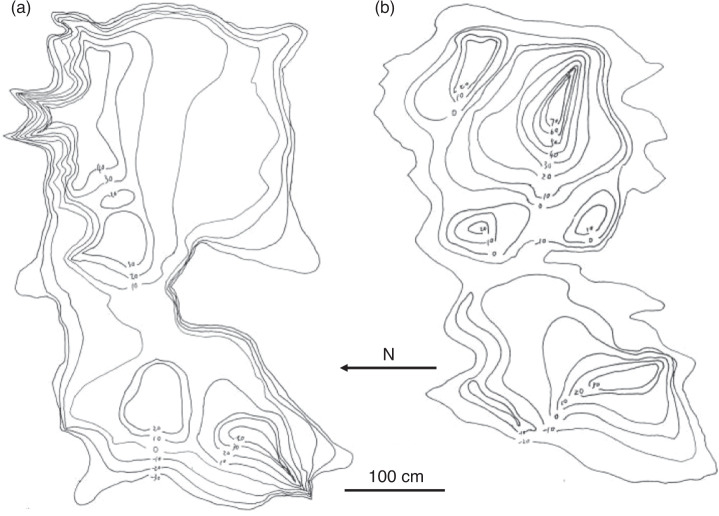
Bathymetric charts of tidepool (**a**) at station HK and (**b**) at station JP.

The bathymetric chart and size of the two tidepools are shown in Fig. 3. At station HK, the distance from the road down to the tidepool is about 200 m. The water volumes of the tidepool were about 4.6 tons at full water level, and about 0.9 ton at low water level. At full water level, the largest depth of the tidepool was about 70 cm, and the average depth was about 30 cm. The tidepool was sized at a ratio of 1:1.86 (largest length:largest width). The areas of the tidepool were about 12.3 m^2^ at full water level, and about 7.8 m^2^ at low water level. The type of tide was a mixed tide dominated by diurnal Tide. At station JP, the distance from the road down to the tidepool is about 400 m. The water volume of the tidepool was about 2.3 tons at full water level, and about 0.8 ton at low water level. At full water level, the largest depth of the tidepool was about 90 cm, and the average depth was about 22 cm. The tidepool was sized at a ratio of 1:1.65 (largest length:largest width). The areas of the tidepool were about 9.3 m^2^ at full water level, and about 4.1 m^2^ at low water level. The type of tide was a mixed tide dominated by semidiurnal Tide.

Fish samples were collected during the four seasons (January to March, April to June, July to September, and October to December) from October 2000 to October 2008 and from April 2012 to November 2018. No samples were collected during 2009–2011 because the project was temporarily suspended and there was no funding for the experiment. Data were collected by counting the number of anesthetized fish during low tide, with an average operation time of approximately 1 h^19^. The sampling days were chosen based on the meteorological conditions obtained from the Central Weather Bureau, Taipei, Taiwan. The anesthetic used in the intertidal fish collection was clove oil mixed with alcohol at a ratio of 1:2 (clove oil:alcohol) and diluted to 100 ppm^19^. The anesthetic was poured into the tidepools and mixed thoroughly. After administering the anesthetic, three laboratory technicians used fishing nets to catch slow-swimming fish. The fish that remained hidden in rock cracks or at the bottom of the pool were carefully collected by hand 15 min later^19^. Fish were placed in sealed packs and transported to the laboratory on ice for identification and counting. The data of individual abundance of fish are absolute values. Fish were identified individually by a fish taxonomist by referring to the Fishbase (http://fishbase.org) and Taiwan Fish Databases (http://fishdb.sinica.edu.tw) and identification keys^30^. Species names were matched with those in the World Register of Marine Species database (https://www.marinespecies.org).

## Data Records

The dataset includes 86 samples collected at tidepools and 43 samples from station HK and station JP, separately. The data are represented as a list of species names and their abundance collected at the two stations^31^. The data contain 82 rows (species) and 86 columns (station, year, and month). The fish assemblage data are provided in a CSV file.

## Technical Validation

**Fig. 4:**
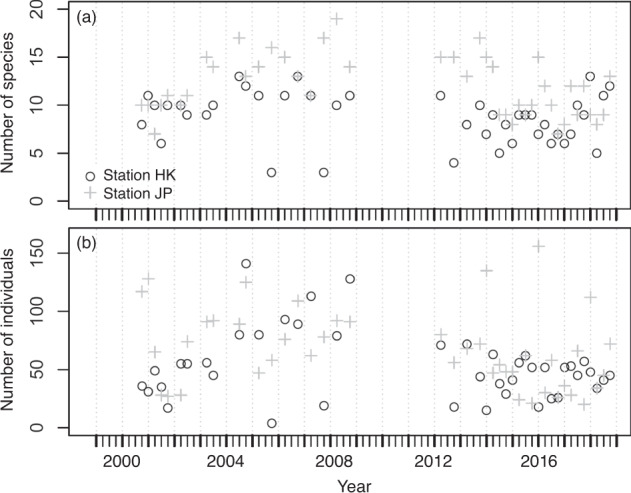
Temporal variations of (**a**) number of species and (**b**) number of individuals at sampling stations during the period 2000–2018.

**Fig. 5:**
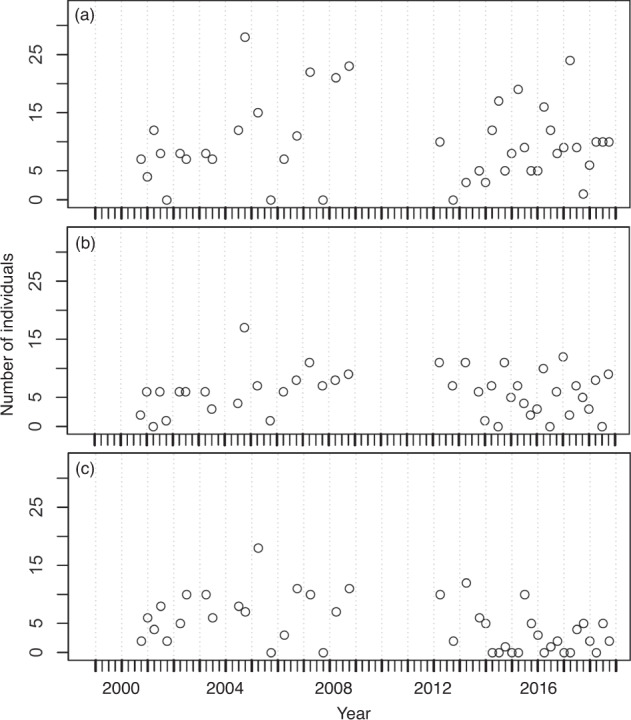
Temporal variations of number of individuals for abundant species at station HK during the period 2000–2018. (**a**) *Istiblennius lineatus*, (**b**) *Istiblennius edentulus*, and (**c**) *Abudefduf sordidus*.

**Fig. 6:**
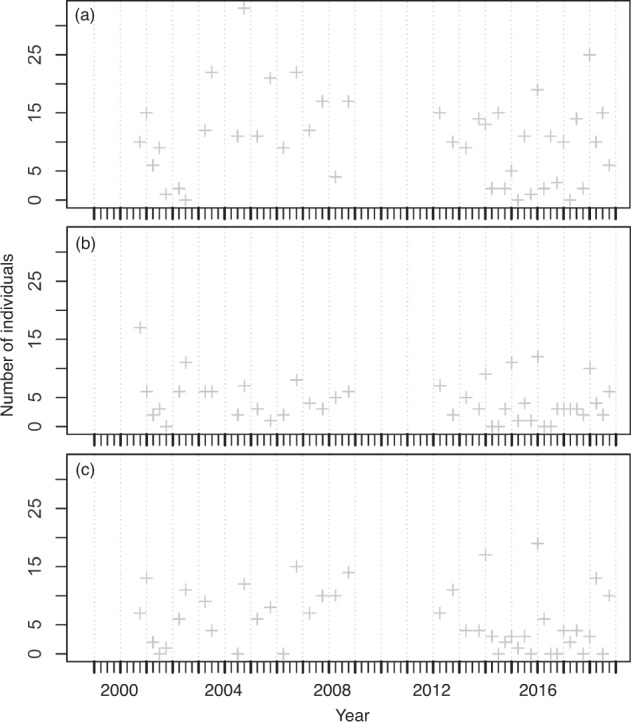
Temporal variations of number of individuals for abundant species at station JP during the period 2000–2018. (**a**) *Istiblennius lineatus*, (**b**) *Abudefduf sordidus*, and (**c**) *Istiblennius edentulus*.

**Fig. 7:**
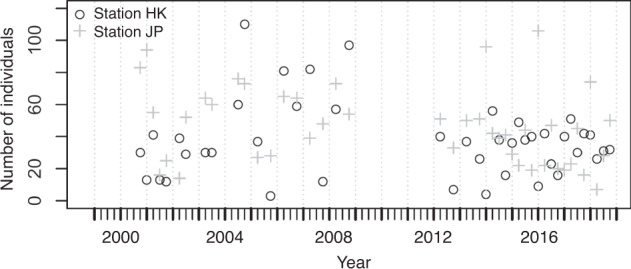
Temporal variations of number of individuals for the species except the three most abundant species at each sampling station during the period 2000–2018.

The number of species and individuals collected at the two stations fluctuated seasonally from 2000 to 2018 and are presented in Fig. 4a,b, respectively. From two stations, 5137 individuals belonging to 82 species were collected. Overall, the number of species collected per sample ranged between 3 and 19 and averaged 10.4 ± 3.3 (s.d.) at the two stations, whereas the number of individuals collected per sample ranged between 4 and 156 and averaged 59.7 ± 32.5 (s.d.). At station HK, 2262 individuals belonging to 53 species were collected. The number of species collected per sample ranged between 3 and 13 with an average of 8.8 ± 2.6 (s.d.), whereas the number of individuals collected per sample ranged between 4 and 141 with an average of 52.6 ± 29.3 (s.d.). At station JP, 2875 individuals belonging to 59 species were collected. The number of species collected per sample ranged between 7 and 19 with an average of 12.0 ± 3.1 (s.d.), whereas the number of individuals collected per sample ranged between 20 and 156 with an average of 66.9 ± 34.2 (s.d.). The most abundant species in the samples were *Istiblennius lineatus*, *I. edentulus*, and *Abudefduf sordidus* at station HK, and *I. lineatus*, *A. sordidus*, and *I. edentulus* at station JP. The long-term temporal fluctuations in the number of individuals of these abundant species for each station are shown in Figs. 5, 6. The seasonal fluctuations in the number of individuals for all species, except for the three most abundant species at stations HK and JP are shown in Fig. 7.

## Data Availability

No custom code was used to generate or process the data described in this manuscript.
